# Yeast processing bodies and stress granules: self-assembly ribonucleoprotein particles

**DOI:** 10.1186/1475-2859-10-73

**Published:** 2011-09-24

**Authors:** Mireia Giménez-Barcons, Juana Díez

**Affiliations:** 1 Department of Experimental and Health Sciences, Universitat Pompeu Fabra, 08003 Barcelona, Spain

**Keywords:** processing bodies, P-bodies, stress granules, RNA granules

## Abstract

Processing bodies (PBs) and stress granules (SGs) are two highly conserved cytoplasmic ribonucleoprotein foci that contain translationally repressed mRNAs together with proteins from the mRNA metabolism. Interestingly, they also share some common features with other granules, including the prokaryotic inclusion bodies. Although the function of PBs and SGs remains elusive, major advances have been done in unraveling their composition and assembly by using the yeast *Saccharomyces cerevisae*.

## Commentary

A growing body of evidence indicates that aggregation of proteins and ribonucleoproteins (RNPs) play a central role in cell biology. It has long been known that cytoplasmic RNP granules containing translationally repressed mRNAs exist in germ cells [1]. Two additional ubiquitous cytoplasmic RNP granules have been recently discovered in somatic cells: the processing bodies (PBs) and the stress granules (SGs) (extensively reviewed in [2-4]). These granules are conserved throughout evolution and are found in yeast, plant, nematode, fly, and mammalian cells. Although they have not yet been observed in prokaryotes, they are found in chloroplasts, organelles of bacterial origin, suggesting that similar structures might also assemble in prokaryotes [5]. PBs contain translationally repressed mRNAs together with proteins from the mRNA decay machinery and, in metazoans, from the miRNA machinery as well. In contrast, SGs contain mRNAs that, although they are also translationally repressed, are stalled in the process of translation initiation, together with translation initiation factors and ribosomal subunits. Both types of granules are highly dynamic and are formed in response to conditions that result in translational repression, including many types of environmental stresses, although PBs are also present in low numbers under normal cell growth [6,7].

Studies in the yeast *Saccharomyces cerevisiae *have been crucial in unraveling PB biology. In yeast, these granules contain a highly conserved set of proteins that belong to the 5' deadenylation-dependent mRNA decay pathway, such as the decapping complex Dcp1/Dcp2, the decapping activators Dhh1, Pat1, Edc3, and Lsm1-7, and the 5'-3'-exonuclease Xrn1p [8]. They also harbor components of the nonsense mediated decay pathway, which rapidly degrades aberrant mRNAs that contain premature stop codons [9]. Since ribosomal subunits are not found in PBs, the mRNPs must be free of ribosomes prior to assemble into PBs [7,10]. Several observations indicate that these mRNAs are also degraded in PBs, since these structures concentrate decapping factors as well as the decay intermediates [11]. However, not all mRNAs that localize in PBs are degraded, as mRNAs have been shown to be able to exit PBs and reinitiate translation [10]. The processes that determine whether an mRNA will be degraded, or sent back into the translation pathway, are not yet understood and are currently the focus of intense research. In addition to ribosome-free mRNAs, two proteins, Edc3 and Lsm4, are also central for PB assembly. Edc3 is a scaffolding protein with a self-aggregation domain, and Lsm4 contains a glutamine/asparagine (Q/N)-rich prion-like domain [12-15]. Similar to the Q/N-rich domains found in prions, the Q/N-rich motif of Lsm4 domain self-aggregates; however, this aggregation is quickly reversible [12].

In contrast to PBs, yeast SGs harbor multiple components of the translation initiation machinery, although their composition varies depending on the type of stress. For example, SGs assembled after glucose deprivation contain eIF4E, eIF4G, Pab1, Pub1, Ngr1 and Pbp1 [16-18], while those induced by severe heat shock contain 40S and eIF3, which are absent from the previous ones [18,19]. The presence of these factors suggests that translationally repressed mRNPs assembled into SGs are stalled at a step in translation initiation that occurs after the recruitment of a subset of the translation initiation machinery [2,4]. Importantly, PBs and SGs interact with each other, probably through shared protein components and mRNA species, and SGs are usually formed either next to or overlapping with PBs [16-19]. These dynamic interactions suggest a cytoplasmic mRNP cycle model in which the mRNAs are exchanged between polysomes, SGs, and PBs, to be translated, stored, or degraded [2,4,18].

Although great advances have been achieved in understanding the composition and assembly of PBs and SGs, their functional significance remains unclear. Elucidating this is especially daunting since basal control of translational repression and mRNA degradation can occur even in the absence of visible PBs and SGs [12,18,20,21]. However, the fact that these granules are evolutionarily conserved strongly suggests that aggregating into larger structures does confer some advantage to the cell, and that these aggregates are functionally important. It has been suggested that aggregates represent a strategy for: i) concentrating enzymes and factors that act successively to optimize the overall processes, ii) sequestering mRNA decay enzymes and thus allowing the decay kinetics to be modulated, and/or iii) preventing repressed mRNAs to compete for the translation machinery [2,3]. Defining these functions is a crucial task that will be of fundamental interest not only for understanding PBs and SGs but also other mRNP granules, since it can be expected that they all function through similar mechanisms.

Formation of microscopic aggregates is not an exclusive function of RNP granules. Many other types of protein granules exist in the cell. For instance, novel and exciting findings show that prokaryotic inclusion bodies (IBs), which were previously believed to be composed solely of misfolded proteins, also contain active polypeptides [22-24]. In eukaryotes, further examples of protein granules are the "purinosome", a multi-enzyme complex in which the enzymes involved in the *de novo *purine biosynthesis dynamically aggregate in response to low purine levels [25,26], and the "eIF2B bodies", in which the translation initiation factors eIF2 and eIF2B are concentrated and which are suggested to be sites of guanine nucleotide exchange [27,28]. In all cases, a dynamic compartmentalization of the cytosol may optimize the function of the aggregated components.

Understanding the still unclear molecular processes leading to the inclusion of mRNPs or proteins into localized foci could have fundamental practical implications in clinical research and in biotechnology. For example, aggresomes, misfolded protein aggregates that are frequently found in neurodegenerative disorders, share some striking similarities with SGs. They share several components and assembly mechanisms, which are mediated by protein-protein aggregation domains. One crucial difference, however, is that SGs are transient and dynamic, whereas aggresomes are static and long-lived [29,30] and references therein]. Interestingly, aggresomes are reminiscent of the prokaryotic IBs. It has recently been reported that IBs can also house self-assembled and highly stable aggregates that have amyloid- or prion-like origins [31]. Thus, bacterial cells could potentially be a valuable tool to study the rules governing protein aggregation in neuronal diseases [32]. On the other hand, the latest advances in artificial engineering of nanoparticles are very promising and have already resulted in highly tunable tools [33,34]. These nanoparticles involve the use of self-assembling peptide sequences to build modular structures that can accommodate a variety of molecules of medical interest. Along this line of reasoning, one could envisage exploiting microbial cell factories to artificially build mRNP aggregates as a way to deliver specific translationally repressed mRNAs, which could then be translated in their recipient cells, thereby tackling diseases by directly modulating protein levels.

## Competing interests

The authors declare that they have no competing interests.

## Authors' contributions

MG and JD have equally contributed to this work. All authors read and approved the final manuscript.
